# Changing Antimicrobial Resistance and Epidemiology of Non-Typhoidal *Salmonella* Infection in Taiwanese Children

**DOI:** 10.3389/fmicb.2021.648008

**Published:** 2021-03-29

**Authors:** Yi-Jung Chang, Yi-Ching Chen, Nai-Wen Chen, Ying-Jie Hsu, Hsiao-Han Chu, Chyi-Liang Chen, Cheng-Hsun Chiu

**Affiliations:** ^1^Department of Pediatrics, Chang Gung Memorial Hospital, College of Medicine, Chang Gung University, Taoyuan, Taiwan; ^2^Molecular Infectious Disease Research Center, Chang Gung Memorial Hospital, Taoyuan, Taiwan; ^3^Graduate Institute of Clinical Medical Sciences, College of Medicine, Chang Gung University, Taoyuan, Taiwan

**Keywords:** antimicrobial resistance, bacteremia, *Salmonella*, antibiotics, children

## Abstract

Non-typhoidal *Salmonella* (NTS) typically causes self-limiting diarrheal disease but may occasionally lead to invasive infection. This study investigated the epidemiology and antimicrobial resistance of children with NTS infection between 2012 and 2019. We retrospectively analyzed pediatric patients with NTS infections, confirmed by positive cultures, in a tertiary medical center in Taiwan in 2012 and 2019. Clinical features and laboratory data of the patients were collected. Changes in the serogroup category and antimicrobial resistance were also analyzed. Of the total 797 isolates collected, 55 had NTS bacteremia. Compared with the resistance rates in 2012, the rates of resistances to third-generation cephalosporin and ciprofloxacin were significantly higher in 2019 (4.1% vs 14.3%, *P* < 0.001; 1.9% vs 28.6%, *P* < 0.001), especially in groups B, D, and E. Moreover, we observed significantly higher antimicrobial resistance (25.3%) to third-generation cephalosporin, and approximately half the NTS isolates in the infant group were multidrug resistant – a higher rate than those of other age groups in 2019. Invasive NTS often presented with a longer fever duration, lower hemoglobin level and with no elevated C-reactive protein (*P* < 0.05). Non-invasive NTS isolates in 2019 were significantly more resistant to ceftriaxone (*P* < 0.001) and ciprofloxacin (*P* < 0.001) than those in 2012. The antimicrobial resistance of NTS in children has increased progressively in the past decade, and different serogroups exhibited different resistance patterns. During this period, infants showed the highest risk to get a third-generation cephalosporin-resistant NTS infection. The high rates of antimicrobial resistance among children with NTS in Taiwan merit continual surveillance.

## Introduction

Non-typhoidal *Salmonella* (NTS) infection is a global public health concern; it results in a considerable disease burden in both industrialized and developing countries (Majowicz et al., 2010; Kyu et al., 2018; Roth et al., 2018; Stanaway et al., 2019). NTS typically causes self-limiting diarrheal disease and may also result in invasive NTS (iNTS) infection (Katiyo et al., 2019). Approximately 5% of individuals with NTS infection develop invasive diseases, such as bacteremia, meningitis, or septic arthritis, which are especially common in young infants or patients with compromised immune systems (Hohmann, 2001; Feasey et al., 2012). Antimicrobial agents are not recommended for non-severe NTS diarrhea, but they are recommended for people at risk of severe or invasive infection (Ke et al., 2020).

Antimicrobial therapy is the first-line therapy for treating patients with NTS with invasive diseases (Tsai et al., 2011). However, the recognition of NTS bacteremia in children before culture is challenging and rarely reported. Furthermore, resistance to antimicrobial agents has increased worldwide, including in Taiwan (Su et al., 2011; James et al., 2018; Chang et al., 2020). Resistant NTS infection has been reported to be correlated with higher morbidity and mortality (Angelo et al., 2016). Therefore, awareness of changes in antimicrobial resistance and the clinical characteristics of NTS is essential for physicians to arrange effective therapeutic plans to prevent complications.

This study investigated the changing trend of antimicrobial resistance and serogroup distribution in children in northern Taiwan between 2012 and 2019 and the risk factors associated with invasive NTS infection.

## Materials and Methods

### Study Population and Data Collection

**FIGURE 1:**
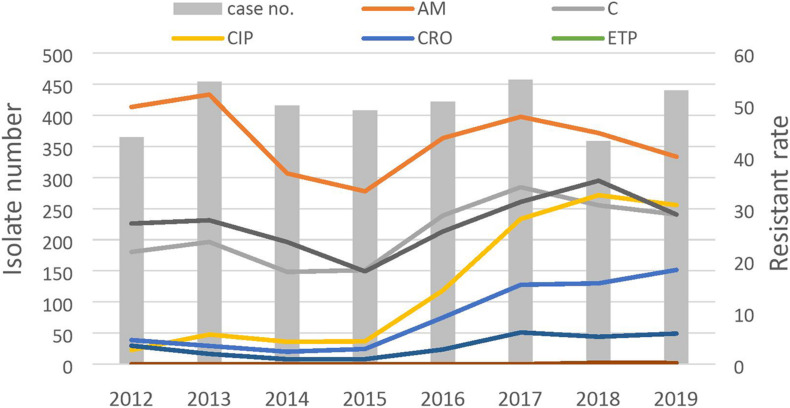
Antibiotic resistant rates of non-typhoidal salmonella from 2012 to 2019.

The secular trend of antimicrobial resistance of NTS in Chang Gung Memorial Hospital is shown in Figure 1. The study enrolled patients aged < 18 years with NTS infection, confirmed by a positive culture, in Chang Gung Memorial Hospital, Linkou, Taiwan, in 2012 and 2019. We retrospectively collected demographic data and clinical characteristics, including patient age, sex, treatment, outcome, serogroup category, and antimicrobial susceptibility from the medical records. In Taiwan, the pediatrician cares patients under 18 of age. In addition, diarrhea is the main cause of morbidity of children under 5 years of age. We stratified participants at different age group, which has been widely validated in many studies. The study was approved by the Research Ethics Committee of Chang Gung Memorial Hospital. (Institutional Review Board Approval No: 201702155B0). All data analyzed were anonymized.

### *Salmonella* Strains and Antimicrobial Susceptibility Testing

We collected stool when the patient had gastrointestinal symptoms. The cerebrospinal fluid was sampled when meningitis is suspected. When a person has symptoms such as urinary irritation or needs to rule out a urinary tract infection, a urine culture would be ordered. At the time of admission in our hospital, each child would have a blood sample taken as usual. The blood samples were sent to microbiology laboratory and incubated in the BACTEC FX automatic blood culture system (Becton Dickinson Diagnostic Instrument Systems, Sparks, MD, United States). The BACTEC FX system monitors the production of carbon dioxide every 10 min and uses a fluorescent signal to indicate positive. The stool samples were planted on HE (Hektoen enteric) and EMB (Eosin Methylene Blue) agar medium, and then incubated at 35°C for 20–24 h. If the same participant had several samples, only 1 sample per patient was collected for downstream analyses. If both blood and stool isolates were available from the same patient, only the blood isolate was analyzed. Our hospital identified each *Salmonella* isolate by using a matrix-assisted laser desorption ionization time-of-flight mass spectrometry automated microbiology system. Serogroups of *Salmonella* isolates were examined with O-antigen antisera (Difco Laboratories, Detroit, MI, United States) by employing the slide agglutination method. We then classified *Salmonella* isolates into five serogroups (group B, C1, C2, D, and E) and other serogroups. The antimicrobial susceptibility of NTS isolates was determined using the Kirby–Bauer disk diffusion method and interpreted according to the Standards for Antimicrobial Susceptibility of the Clinical Laboratory Standards Institute. The CLSI versions M100-S29 in 2019 and M100-S22 in 2012 were used in this study (Clinical and Laboratory Standards Institute [CLSI], 2012, 2019). The following antimicrobial agents were examined: ciprofloxacin, ceftriaxone, ampicillin, chloramphenicol, ertapenem, imipenem, flomoxef, and trimethoprim-sulfamethoxazole (TMP-SMX). In MIC determination, the concentration of ceftriaxone resistant was with MIC 4 μg/mL and intermediate was with MIC 1–4 μg/mL. Meanwhile, the concentration of ciprofloxacin resistant was with MIC 1 μg/ml and intermediate was with MIC 0.5 μg/ml. The MIC range of chloramphenicol was 2.0–8.0 μg/mL. For further statistical analysis, intermediate resistance was regarded as the threshold for resistance. Multi-drug resistance (MDR) was defined as concomitant resistance to ≥three antimicrobial drug classes with the same selection, including ampicillin, ceftriaxone, chloramphenicol, ciprofloxacin, imipenem, TMP-SMX, ertapenem, and flomoxef. To analyze factors associated with invasive NTS in children, patients with *Salmonella* bacteremia, meningitis, or septic arthritis and without invasive salmonellosis were analyzed. For clinical features, only children who had both blood and stool culture performed were analyzed. We further divided the study patients into two groups. Those with diarrhea and negative blood culture were categorized as the non-invasive group. Those had positive results either in blood, CSF, or pus culture would be categorized into the invasive group. Patient eligibility was determined according to factors such as clinical manifestations and laboratory data obtained upon admission.

### Statistical Analysis

We completed statistical analysis using SPSS for Windows version 22.0 (SPSS Inc., Chicago, IL, United States). Categorical variables including multiple categories were analyzed using the chi-square test, and continuous variables were examined using independent *t* tests. A *p* value <0.05 was considered to be statistically significant.

## Results

A total of 797 NTS isolates were collected from 781 patients in 2012 and 2019 (363 from 2012 and 434 from 2019), of which 90.7% were isolated from the stool, 6.8% from blood, and 2.5% from other extraintestinal samples (12 isolates from urine, 3 from cerebrospinal fluid, and 2 from pus). The isolates collected from blood and extraintestinal samples were classified as iNTS. The majority (81.5%) of the NTS isolates were obtained from children aged ≤ 3 years, and 36.0% of the isolates were from children aged 1–2 years. The most common serogroup was group D (42.6%), followed by group B (24.8%), serogroup C2 (12.6%), and serogroup E (12.1%). The overall resistance rates were 15.1% to ciprofloxacin, 8% to ceftriaxone, 41.4% to ampicillin, 27.8% to chloramphenicol, 2.3% to ertapenem, 3.9% to flomoxef, 0.6% to imipenem, and 28.2% to sulfamethoxazole. Table 1 presents a comparison of the baseline characteristics of the patients and antimicrobial resistance rates of NTS between 2012 and 2019. The mean age of the two groups was similar (2.1 years vs 2.4 years, *P* = 0.56). Compared with the resistance rates in 2012, the resistance rates in 2019 to ciprofloxacin (28.6% vs 1.9%, *P* < 0.001), ceftriaxone (14.3% vs 4.1%, *P* < 0.001), chloramphenicol (31.3% vs 21.7%, *P* = 0.003), and sulfamethoxazole (29.4% vs 27.2%, *P* < 0.001) were significantly higher. Moreover, the ampicillin resistance rate was significantly lower in 2019 than in 2012 (49.2% vs 40.1%, *P* = 0.01). No significant difference in resistance rate was observed for ertapenem, imipenem, and flomoxef between those 2 years. The incidence of ampicillin as an empirical antibiotic in 2012 was higher than in 2019 (18.8% vs 4.1%, *P* < 0.001). On the contrary, if directed, most doctors choose ceftriaxone as an empirical antibiotic in 2019 than in 2012 (79.5% vs 56.9%, *P* < 0.001). The evolution of antimicrobial resistance in different serogroups is presented in Table 2. The highest resistance rate to ampicillin was noted in group B (48%), and the highest resistance rate to ciprofloxacin was observed in group C1 (27.1%). We noted significantly increased ciprofloxacin, ceftriaxone, and MDR resistance between 2012 and 2019 in the isolates from group B, group D, and group E. In groups C1 and C2, we also noted an increase in ciprofloxacin resistance in 2019 compared with 2012. Table 3 presents the differences in antimicrobial resistance by age for 2012 and 2019. In 2019, the patients aged < 1 year had a higher resistance rate to ampicillin (50.6%, *P* = 0.005), ceftriaxone (25.3%, *P* = 0.002), TMP-SMX (44.2%, *P* = 0.001), and chloramphenicol (46.3%, *P* = 0.003) than did that other age group. The differences in clinical manifestations and laboratory examination results upon admission between the NTS bacteremia group and the non-iNTS group are listed in Table 4. Longer fever duration (3.8 ± 2.7, *P* < 0.001), lower C-reactive protein (CRP) level (39.1 ± 41.8 vs 58.6 ± 55.7, *P* < 0.001), and lower hemoglobin level (11.6 ± 1.4 vs 12.1 ± 1.2, *P* = 0.027) were significant factors associated with the NTS bacteremia group. The main outcomes between children with iNTS and those with diarrhea and a negative blood culture was the prolonged hospital stay in iNTS (9.8 ± 9.5 vs 5.8 ± 12.1 *p* = 0.016). Table 5 presents the antimicrobial resistance in NTS bacteremia and non-iNTS infections in 2012 and 2019. Non-iNTS isolates in 2019 were significantly more resistant to ceftriaxone (15.1% vs 3.8%, *P* < 0.001) and ciprofloxacin (28.5% vs 1.7%, *P* < 0.001) than those in 2012.

**TABLE 1:** Baseline characteristics and clinical features of 797 patients with non-typhoid *Salmonella* infection in 2012 and 2019.

*N* (%)	2012 (*N* = 363)	2019 (*N* = 434)	*P* value
Males (n, %)	200 (55.1%)	239 (55.1%)	0.994
Age(mean) (y/o)	2.1 (SD = 3.8)	2.4 (SD = 3.8)	0.56
Admission proportion	74.2%	62.7%	<0.001
Admission duration (days)	6.9 ± 16.2	5.4 ± 5.9	0.139
Ampicillin use	27/144(18.8%)	8/195 (4.1%)	<0.001
Ceftriaxone use	82/144(56.9%)	155/195(79.5%)	<0.001
iNTS	23/364 (6.3%)	39/434 (7.9%)	0.123
***Salmonella* serogroups**			
Group B	98/364 (26.9%)	100/434 (23%)	0.206
Group C1	20/364 (5.5%)	29/434 (6.7%)	0.486
Group C2	45/364 (12.4%)	56/434 (12.9%)	0.819
Group D	155/364 (42.6%)	185/434 (42.6%)	0.990
Group E	40/364 (11%)	57/434 (13.1%)	0.356
**Antimicrobial resistance**			
MDR	44/364 (12.2%)	130/434 (30%)	<0.001
Ampicillin	179/364 (49.3%)	174/434 (40.1%)	0.009
Ceftriaxone	15/364 (4.1%)	62/434 (14.3%)	<0.001
Ciprofloxacin	7/364 (1.9%)	124/433 (28.6%)	<0001
TMP-SMX	99/364 (27.3%)	127/432 (29.4%)	0.508
Ertapenem	0/364 (0%)	3/434 (0.7%)	0.255
Imipenem	0/364 (0%)	5/434 (1.2%)	0.067
Chloramphenicol	79/364 (21.8%)	126/403 (31.3%)	0.003
Flomoxef	12/364 (3.3%)	24/403 (6%)	0.084

*iNTS, invasive non-typhoidal *Salmonella*; MDR, multi-drug resistance, resistant to ≥3 drug classes; TMP-SMX: trimethoprim-sulphamethoxazole.*

**TABLE 2:** Changing antimicrobial resistance in different serogroup between 2012 and 2019.

Antimicrobials	2012	2019	*P* value	OR	95% CI
**Serogroup B**					
MDR	11.2% (11/98)	32% (32/100)	<0.001	3.72	1.74−7.91
Ceftriaxone	5.1% (5/98)	15% (15/100)	0.021	3.28	1.14−9.41
Ciprofloxacin	1% (1/98)	27.3% (27/99)	<0.001	36.3	4.83−273.95
Ampicillin	53.1% (52/98)	43% (43/100)	0.157	1.49	0.85−2.62
TMP-SMX	26.5% (26/98)	35% (35/100)	0.197	0.671	0.365−1.232
Ertapenem	0% (0/98)	1% (1/100)	1.000	NA	NA
Imipenem	0% (0/98)	2% (2/100)	0.498	NA	NA
**Serogroup C1**					
MDR	10% (2/20)	34.5% (10/29)	0.089	4.73	0.91−24.64
Ceftriaxone	0% (0/20)	20.7% (6/29)	0.069	NA	NA
Ciprofloxacin	0% (0/20)	44.8% (13/29)	<0.001	NA	NA
TMP-SMX	26.3% (5/19)	32.1% (9/28)	0.668	0.754	0.20−2.74
Ampicillin	47.4% (9/19)	44.8% (13/29)	0.863	1.10	0.34−3.53
Ertapenem	0% (0/19)	3.4% (1/29)	1.000	NA	NA
Imipenem	0% (0/19)	3.4% (1/29)	1.000	NA	NA
**Serogroup C2**					
MDR	8.9% (4/45)	16.1% (9/56)	0.284	1.96	0.56−6.85
Ceftriaxone	4.4% (2/45)	5.4% (3/56)	1.000	1.21	0.19−7.61
Ciprofloxacin	2.2% (1/45)	19.6% (11/56)	0.007	10.75	1.33−86.85
TMP-SMX	31.1% (14/45)	14.3% (8/56)	0.042	2.70	1.01−7.21
Ampicillin	62.6% (28/45)	23.2% (13/56)	<0.001	5.44	2.29−12.93
Ertapenem	0% (0/45)	0% (0/56)	NA	NA	NA
Imipenem	0% (0/45)	0% (0/56)	NA	NA	NA
**Serogroup D**					
MDR	14.2% (22/155)	31.4% (58/185)	<0.001	2.76	1.59−4.77
Ceftriaxone	5.2% (8/155)	15.1% (28/185)	0.003	3.27	1.44−7.42
Ciprofloxacin	2.6% (4/155)	27.6% (51/185)	<0.001	14.36	5−40.8
TMP-SMX	27.7% (43/155)	30.4% (56/184)	0.587	0.878	0.548−1.406
Ampicillin	45.8% (71/155)	43.2% (80/185)	0.636	1.10	0.72−1.70
Ertapenem	0% (0/155)	0% (0/185)	NA	NA	NA
Imipenem	0% (0/155)	0.5% (1/185)	1.000	NA	NA
**Serogroup E**					
MDR	12.5% (5/40)	31.6% (18/57)	0.030	3.23	1.08−9.61
Ceftriaxone	0% (0/40)	14% (8/57)	0.019	NA	NA
Ciprofloxacin	2.5% (1/40)	19.6% (19/57)	<0.001	19.5	2.48−152.98
TMP-SMX	27.5% (11/40)	28.1% (16/57)	0951	0.972	0.394−2.398
Ampicillin	42.5% (17/40)	38.6% (22/57)	0.700	1.17	0.51−2.67
Ertapenem	0% (0/40)	0% (0/57)	NA	NA	NA
Imipenem	0% (0/40)	0% (0/57)	NA	NA	NA

*MDR, multi-drug resistance, resistant to ≥3 drug classes; TMP-SMX, trimethoprim/sulfamethoxazole; OR, odds ratio; CI, confidence interval; NA, not applicable.*

**TABLE 3:** Changing antimicrobial resistance by different age groups between 2012 and 2019.

AGE	<1 Y	1–2 Y	2–5 Y	>5 Y	*P* value
**2012**					
MDR	12.1%(12/99)	11.6%(18/155)	14.9%(14/94)	0%(0/15)	0.426
Ampicillin	48.5%(48/99)	54.2%(84/155)	42.3%(22/52)	43.1%(25/58)	0.279
Ceftriaxone	3%(3/99)	5.2%(8/155)	4.3%(4/94)	0%(0/15)	0.575
Ciprofloxacin	2%(2/99)	1.9%(3/155)	2.1%(2/94)	0%(0/15)	0.895
TMP-SMX	25.3%(25/99)	25.8%(40/155)	34%(32/94)	13.3%(2/15)	0.260
Ertapenem	0%(0/99)	0%(0/155)	0%(0/94)	0%(0/15)	
Imipenem	0%(0/99)	0%(0/155)	0%(0/94)	0%(0/15)	
Chloramphenicol	28.3%(28/99)	22.6%(35/155)	16%(15/94)	6.7%(1/15)	0.094
Flomoxef	4%(4/99)	2.6%(4/155)	4.3%(4/94)	0%(0/15)	0.641
**2019**					
MDR	42.5%(37/87)	22.2%(40/180)	29.6%(37/125)	38.1%(16/42)	0.005
Ampicillin	50.6%(44/87)	32.8%(59/180)	41.6%(52/125)	19.0%(8/42)	0.036
Ceftriaxone	25.3%(22/87)	8.3%(15/180)	13.6%(17/125)	19.0%(8/42)	0.002
Ciprofloxacin	34.9%(30/86)	23.9%(43/180)	28.8%(36/125)	35.7%(15/42)	0.199
TMP-SMX	44.2%(38/86)	22.3%(40/179)	26.4%(33/125)	38.1%(16/42)	0.001
Ertapenem	2.3%(2/87)	0.6%(1/180)	0%(0/125)	0%(0/42)	0.221
Imipenem	3.4%(3/87)	0.6%(1/180)	0%(0/125)	2.4%(1/42)	0.084
Chloramphenicol	46.3%(37/80)	24.4%(39/160)	27.9(34/122)	39%(16/41)	0.003
Flomoxef	3.8%(3/80)	4.4%(7/160)	8.2%(10/122)	49.8%(4/41)	0.326

*MDR, multi-drug resistance, resistant to ≥3 drug classes.*

**TABLE 4:** Demographic, clinical manifestations and antimicrobial resistance among iNTS and non-iNTS infections in 2012 and 2019.

	iNTS infections (*n* = 62)	Non-iNTS infections (*n* = 555)	*P* value
Male, *n* (%)	53.2% (33)	56.5% (314)	0.582
Age, y/o, mean ± SD	1.7 ± 2.8	2.3 ± 2.3	0.099
**Symptoms at admission**			
Fever, *n* (%)	58(93.5%)	512(92.8%)	0.930
Diarrhea, *n* (%)	45(72.6%)	376(68.1%)	0.444
Bloody stools, *n* (%)	19(43.2%)	242(58%)	0.059
Abdominal pain, *n* (%)	9(14.5%)	186(33.7%)	0.001
Vomiting, *n* (%)	18(29%)	208(37.3%)	0.087
Fever duration, days, mean ± SD	3.8 ± 2.4	2.7 ± 2.1	< 0.001
Hospital stay, days, mean ± SD	9.8 ± 9.5	5.8 ± 12.1	0.016
Hb, g/dL, mean ± SD	11.6 ± 1.4	12.1 ± 1.2	0.027
CRP, mg/L, mean ± SD	39.1 ± 58.3	58.6 ± 54.8	0.009
Band form, %, mean ± SD	4.7 ± 6.9	6.9 ± 6.9	0.112
WBC, /μl, mean ± SD	10,803 ± 4,420	10,051 ± 4,168	0.192
**Antimicrobial resistance**			
**2019**			
Ampicillin	29.7%	41.6%	0.165
Ceftriaxone	5.4%	14.8%	0.119
Ertapenem	2.7%	0.3%	0.209
Imipenem	2.8%	1.0%	0.353
Ciprofloxacin	30.6%	28.5%	0.790
TMP-SMX	20.6%	30.2%	0.240
Chloramphenicol	66.7%	29.9%	0.073
Flomoxef	40%	5.5%	0.030
**2012**			
Ampicillin	42.1%	49.7%	0.519
Ceftriaxone	10.5%	3.8%	0.182
Ertapenem	0%	0%	NA
Imipenem	0%	0%	NA
Ciprofloxacin	5.3%	1.7%	0.316
TMP-SMX	15.8%	27.9%	0.248
Chloramphenicol	21.1%	21.8%	1.000
Flomoxef	5.3%	3.2%	0.624

*iNTS, invasive non-typhoidal *Salmonella*; SD, standard deviation; TMP-SMX, trimethoprim-sulfamethoxazole.*

**TABLE 5:** Antimicrobial resistance and serogroups between iNTS and non-iNTS infections in 2012 and 2019.

	iNTS			non-iNTS		
	2012	2019	*P* value	2012	2109	*P* value
**Antimicrobial resistance**						
Ampicillin	42.1%	27.8%	0.282	49.7%	41.2%	0.020
Ceftriaxone	10.5%	5.6%	0.602	3.8%	15.1%	<0.001
Ertapenem	0%	2.8%	1.000	0%	0.5%	0.502
Imipenem	0%	2.8%	1.000	0%	1.0%	0.128
Ciprofloxacin	5.3%	30.6%	0.041	1.7%	28.5%	<0.001
TMP-SMX	15.8%	20.6%	1.000	27.9%	30.2%	0.518
Chloramphenicol	21.2%	60%	0.126	21.8%	30.9%	0.005
Flomoxef	5.3%	40%	0.099	3.2%	5.5%	0.125
MDR	10.5%	13.9%	1.000	12.2%	31.4%	<0.001
**Serogroups**						
B	15.8%	22.2%	0.730	27.6%	23.1%	0.159
C1	15.8%	2.8%	0.114	4.7%	7%	0.170
C2	5.3%	16.7%	0.401	12.8%	12.6%	0.926
D	52.6%	41.7%	0.437	42.2%	42.7%	0.891
E	10.5%	13.9%	1.000	11%	13.1%	0.401

*iNTS, invasive non-typhoidal *Salmonella*; MDR, multi-drug resistance, resistant to ≥3 drug classes; TMP-SMX, trimethoprim-sulfamethoxazole.*

## Discussion

Our results indicated that the antimicrobial susceptibility of NTS has changed during the past decade, with several clinically relevant findings. A particularly crucial finding is the significantly higher prevalence of antimicrobial resistance to third-generation cephalosporin in infants. Additionally, the resistance rate to ciprofloxacin and third-generation cephalosporin increased significantly from 2012 to 2019, especially in groups B, D, and E. Furthermore, we observed that most patients with NTS bacteremia presented with fever lasting more than 4 days at admission with a lower systemic inflammatory reaction. This is the first analysis to reveal that *Salmonella* bacteremia is closely associated with longer fever duration and inconsistent lower systemic inflammatory reaction. By contrast, non-blood isolates (mostly local) were generally more resistant than NTS bacteremia isolates.

Concern has been growing over the past decades regarding antimicrobial resistance in NTS. Our data demonstrated an increase in overall antimicrobial resistance in *Salmonella* from 20 to 30% in 2012 to as high as 70% in 2019. Previous studies have reported high resistance rates to ampicillin in Taiwan. The increasing rates of resistance have resulted in changes to clinical practice guidelines for treating the disease. Therefore, expensive third-generation cephalosporins are currently the first-choice antibiotics for iNTS infection in children. Our data revealed a significant elevation in ceftriaxone resistance from 2012 to 2019, whereas the resistance rate to ampicillin decreased. These results are in accordance with recent studies indicating the emergence and spread of resistance to third-generation cephalosporin in Taiwan and other Asian countries (Lee et al., 2021). Ampicillin resistance significantly decreased in our study period, possibly because of antibiotic choice.

This study explored various NTS serogroups that are closely associated with antimicrobial resistance. More than half the NTS isolates were classified as MDR, with particularly high resistance in groups B, D, and E. These three groups also had the highest resistance rate to third-generation cephalosporin. In Taiwan, self-transferable *bla*_CMY–__2_-harboring IncI1 plasmids have been identified in several *Salmonella* serotypes belonging to *Salmonella* groups B and D (Lo et al., 2020). Since 2015, northern Taiwan has experienced an increase in *Salmonella* infections caused by previously rare *Salmonella* Anatum, a serogroup E serotype. Such infections have also been reported in central Taiwan, indicating that this outbreak has already spread throughout the island (Chiou et al., 2019). Coresistance to ceftriaxone and ciprofloxacin is the main feature of the outbreak clone (Feng et al., 2020).

This study highlighted that isolates from infants had significantly higher MDR to ampicillin and third-generation cephalosporin than did isolates from other age groups. This is partly explained by the increase in isolates in infants with serotypes since the early 1990s, which have been associated with MDR (Katiyo et al., 2019). This rather unexpected result may be attributed to human-to-human transmissions. Parents may have more often touched, rinsed, and cooked contaminated meat before feeding other foods to their infants. Moreover, these parents were more willing to purchase meat from traditional markets rather than supermarkets (Feng et al., 2020). One possibility is that parents bought meat from traditional markets, and then, their frequent rinsing spread the *Salmonella* from the surface of the meats to cutting boards, knives, sinks, and finally onto fresh vegetables, fruit, and other ready-to-eat foods that were cross-contaminated and reached the infants through parents or other caregivers. This transmission mode is particularly critical in infants. On the basis of these findings, we recommend that clinicians in Taiwan use third-generation cephalosporins for empirical treatment of NTS bacteremia and determine the exact treatment according to antimicrobial susceptibility results.

Our analyses indicated that ceftriaxone resistance in Taiwan was low in 2012, and our results revealed that the overall resistance rates in the non-iNTS isolates were generally higher than those in the iNTS isolates. We noted significantly lower serum CRP level in patients with NTS bacteremia than in patients without NTS bacteremia. In previous studies, a severity score with CRP served as a guide for prescribing antibiotics for severe NTS infection in children. On the basis of the current findings, we recommend against routinely using antimicrobials to treat uncomplicated NTS gastroenteritis in cases of high CRP level. This finding is consistent with that of Mughini-Gras et al. (2020) who reported that iNTS isolates were generally less resistant than non-iNTS isolates (Mughini-Gras et al., 2020; Ke et al., 2020). NTS bacteremia in this study tended to develop in young children with prolonged fever. The hemoglobin level was also found significantly lower in the invasive group (*P* = 0.027) in our study. Christenson (2013) had reported that patients with hemolytic anemia, malaria, sickle cell anemia, and thalassemia major were more susceptible to invasive *Salmonella* infection (Christenson, 2013). This finding is consistent with previous reports that revealed a longer duration of symptoms, such as fever and diarrhea, before admission in children with bacteremia (Lee et al., 2021). Thus, fever duration can be a suitable rationale for treatment before receiving antimicrobial susceptibility results.

Adopting an 8-year detailed hospitalization dataset from the largest pediatric hospital in Taiwan enabled us to representatively evaluate the trends and characteristics of antimicrobial resistance in pediatric NTS infections. However, this study has some limitations. First, only serogroup data were available in our research, and serotype data were limited. Disease severity and antimicrobial resistance may be associated with different serotypes; therefore, further analysis of serotypes with clinical manifestations or resistance patterns are required to clarify virulence and impact among different serotypes. Second, this was a single-center study; thus, we could not adequately account for the considerable geographic variability in antimicrobial resistance patterns in NTS. Although the data is from a single hospital, Chang Gung Memorial Hospital is the largest in Taiwan. So, the data can reflect the true epidemiology in Taiwan.

This study revealed that the resistance of NTS to fluoroquinolones and third-generation cephalosporins has been increasing in Taiwan. MDR in serogroups B, D, and E remained high. More importantly, cephalosporin resistance was more common in isolates from infants. Prolonged fever was a single factor associated with bacteremia in children with NTS infection.

## Data Availability Statement

The original contributions presented in the study are included in the article/supplementary material, further inquiries can be directed to the corresponding author/s.

## Author Contributions

C-HC and Y-JC: conception and design of the study. N-WC and Y-JH: implementation and data collection. H-HC and Y-CC: analysis and interpretation of the data. Y-JC, C-LC, and C-HC: writing and critical review of the manuscript. All authors contributed to the article and approved the submitted version.

## Conflict of Interest

The authors declare that the research was conducted in the absence of any commercial or financial relationships that could be construed as a potential conflict of interest.
